# Face recognition ability does not predict person identification performance: using individual data in the interpretation of group results

**DOI:** 10.1186/s41235-018-0117-4

**Published:** 2018-06-27

**Authors:** Eilidh Noyes, Matthew Q. Hill, Alice J. O’Toole

**Affiliations:** School of Behavioral and Brain Sciences, GR4.1, The University of Texas at Dallas, 800 W Campbell Road, Richardson, TX 75080-3021 USA

**Keywords:** Face recognition, Body recognition, Biological motion, Individual differences, Pre-screening

## Abstract

There are large individual differences in people’s face recognition ability. These individual differences provide an opportunity to recruit the best face-recognisers into jobs that require accurate person identification, through the implementation of ability-screening tasks. To date, screening has focused exclusively on face recognition ability; however real-world identifications can involve the use of other person-recognition cues. Here we incorporate body and biological motion recognition as relevant skills for person identification. We test whether performance on a standardised face-matching task (the Glasgow Face Matching Test) predicts performance on three other identity-matching tasks, based on faces, bodies, and biological motion. We examine the results from group versus individual analyses. We found stark differences between the conclusions one would make from group analyses versus analyses that retain information about individual differences. Specifically, tests of correlation and analysis of variance suggested that face recognition ability was related to performance for all person identification tasks. These analyses were strikingly inconsistent with the individual differences data, which suggested that the screening task was related only to performance on the face task. This study highlights the importance of individual data in the interpretation of results of person identification ability.

## Significance statement

Screening tasks are now being implemented in the recruitment of candidates for jobs that involve person identification. To date, such screening has focussed exclusively on face-recognition ability. However, real-world identifications often involve the use of cues from the face, the body, and biological motion. We test whether screening on face-recognition ability predicts person-identification performance more broadly. This research has important implications for the applied field of person recognition.

## Background

Human face-recognition ability varies widely from person to person. People who perform with exceptionally high accuracy on face-recognition tasks are called “super”-recognisers (see Noyes, Phillips, & O’Toole, 2017 for a review). People with prosopagnosia are at the other end of the spectrum—these people experience severe difficulty with face recognition (see Kress & Daum, 2003). The ability of the rest of the population is dispersed between these two extremes. Many jobs require accurate identifications to be made for security and legal purposes. Screening candidates for these jobs on their person-identification abilities would, in theory, create a workforce of people best skilled for the job. To date, screening has focused exclusively on *face* recognition. However, real-world identification scenarios often include other information that can aid identification such as the body or a person’s movement.

More broadly, research suggests that the body provides information that can aid identification. Accurate identifications are made more frequently from images of faces, than images of the body (Burton, Wilson, Cowan, & Bruce, 1999; O'Toole et al., 2011; Robbins & Coltheart, 2012). Despite this, above-chance accuracy has been achieved on matching tasks that involve pairs of body images (O'Toole et al., 2011). Additionally, fusing identification decisions from the face, with those from the body, provided more accurate identity decisions than from the face alone (O'Toole et al., 2011). The role of the body in identifications is supported further by Robbins and Coltheart (2015), who reported that participants made more accurate identification decisions from full-person stimuli (video footage or static image) over face-only and body-only stimuli. It follows that people rely more on the face than the body to inform their identity judgements (Robbins & Coltheart, 2012), even when they identify familiar people (Burton et al., 1999). However, when it is difficult to extract information from the face, the body can be relied upon to inform identification, even without conscious awareness of this reliance (Hahn, O’Toole, & Phillips, 2016; Rice, Phillips, Natu, An, & O’Toole, 2013).

A person’s movement can also facilitate identification (O'Toole et al., 2011; Robbins & Coltheart, 2015; Simhi & Yovel, 2016). O'Toole et al. (2011) reported that participants achieved higher matching accuracy when they viewed video footage of people’s bodies (face obscured), than when they viewed static body images. Conversely, Robbins and Coltheart (2015) reported no benefit of movement for person recognition on images that showed the face only, the body only, or the full person. These studies tested for the benefits of movement using natural videos of faces and bodies. Often, studies that have examined the role of motion in recognition use point light biological motion to isolate movement from an image of a body. These point light videos were originally created by attaching lights, florescent tape, or markers to the joints of people who are dressed in dark clothing, and then filming movements of these people in a dark room (see Johansson, 1973). They can be created now with computer software by attaching markers to the joints and key reference points of models (e.g. head, arms, legs, shoulders, elbows, knees). Identity information can be extracted from biological motion, with studies showing that familiar people can be identified from point light motion videos (Barclay, Cutting, & Kozlowski, 1978; Beardsworth & Buckner, 1981; Cutting & Kozlowski, 1977; Jacobs & Shiffrar, 2004; Loula, Prasad, Harber, & Shiffrar, 2005). There is also evidence of unfamiliar-person learning and matching from point light motion displays (Baragchizadeh & O’Toole, 2017; Loula et al., 2005; Stevenage, Nixon, & Vince, 1999; Troje, 2005).

Here we asked whether a standard screening test of face recognition ability can be used to predict a person’s ability to make identifications from the face, the body, and biological motion. To date, there is only one study that has tested for an association between face-recognition ability and body-recognition ability (Biotti, Gray, & Cook, 2017). In that study, participants with prosopagnosia and control subjects were tested on face and body recognition skill on static images (Biotti et al., 2017). As a group, people with prosopagnosia were significantly worse than controls at recognising images of faces and bodies. Scatterplots highlighted that some, but not all, prosopagnosic participants were impaired at body recognition. We are interested in the relationship between face recognition, body recognition, and recognition from biological motion across the spectrum of face-recognition ability encountered in the general population.

In generating hypotheses about the relationship between face-recognition accuracy and accuracy on other person-identification tasks, we can consider the use of processing strategies. For example, previous studies show that people recruit similar holistic processing strategies when they view face images (Collishaw & Hole, 2000; Maurer, Le Grand, & Mondloch, 2002; Murphy, Gray, & Cook, 2017; Rossion, 2013; Tanaka & Simonyi, 2016; Tanaka & Farah, 1993; Young, Hellawell, & Hay, 1987), body images (Aviezer & Todorov, 2012; Robbins & Coltheart, 2012; Seitz, 2002), and biological motion videos (Bertenthal & Pinto, 1994; Chatterjee, Freyd, & Shiffrar, 1996; Thompson, Clarke, Stewart, & Puce, 2005). Moreover, similar inversion effects have been found for face, body, and full-person images (Minnebusch, Suchan, & Daum, 2008; Reed, Stone, Bozova, & Tanaka, 2017; Robbins & Coltheart, 2012; Yovel, Pelc, & Lubetzky, 2010). However, other studies have reported no inversion effects for headless bodies (Minnebusch et al., 2008; Robbins & Coltheart, 2012; Yovel et al., 2010) and Bauser, Suchan, and Daum (2011) found no evidence of integration of the top and bottom half of body-only images. Inversion effects have been reported also for biological-motion stimuli (Pavlova & Sokolov, 2000; Sumi, 1984; Troje & Westhoff, 2006).

A different hypothesis may be generated from an examination of the neural processes activated by the face, body, and movement. Despite the similarities in processing strategies described above, distinct neural processes are activated by face and body images (Kanwisher & Yovel, 2006; Peelen & Downing, 2007). A complex cortical network of brain regions is involved in face recognition (Calder & Young, 2005; Haxby, Hoffman, & Gobbini, 2000), which includes the fusiform face area (FFA) (Gobbini & Haxby, 2007; Grill-Spector, Knouf, & Kanwisher, 2004; Kanwisher, Mcdermott, & Chun, 1997; Kanwisher & Yovel, 2006) and the occipital face area (OFA) (Pitcher, Walsh, & Duchaine, 2011; Pitcher, Walsh, Yovel, & Duchaine, 2007). Body recognition has been linked to activation of the extra-striate body area (EBA) and the fusiform body area (FBA) (Downing, 2001; Kanwisher & Yovel, 2006) in the ventral visual stream. Furthermore, the posterior superior temporal sulcus (pSTS) in the dorsal visual stream is activated by viewing motion of the face, motion of the body, and more generally, biological motion (Allison, Puce, & McCarthy, 2000; Beauchamp, Lee, Haxby, & Martin, 2003; Fox, Iaria, & Barton, 2009; Giese & Poggio, 2003; Gilaie-Dotan, Kanai, Bahrami, Rees, & Saygin, 2013; Yovel & O’Toole, 2016). The distributed model of Haxby et al. (2000) predicts that the invariant information about faces and bodies is processed by ventral stream areas (FFA, OFA, FBA, and EBA). The changeable information from biological motion is processed by the dorsal stream regions in the pSTS. If recognition abilities reflect the neural processing systems, face and body processing ability may be linked, while biological motion processing could be independent of these other abilities.

Turning now to the methodological questions of how skills are related, there are two main challenges inherent in any investigation of the relationship among person recognition skills. First, there is the challenge of incorporating individual differences into conclusions. For example, the literature depicts super-recognisers as consistent high performers across a range of face-recognition tasks (Bobak, Bennetts, Parris, Jansari, & Bate, 2016; Bobak, Dowsett, & Bate, 2016; Bobak, Hancock, & Bate, 2016; Davis, Lander, Evans, & Jansari, 2016; Noyes et al., 2017; Robertson, Noyes, Dowsett, Jenkins, & Burton, 2016; Russell, Duchaine, & Nakayama, 2009). This conclusion most often reflects group-level results (Noyes et al., 2017). In this literature, group-level results most often compare the average performance of super-recognisers on a task against the average performance of control participants on the same task. At a group level, super-recognisers outperform control groups on a range of face-recognition tasks. However, there are often complex patterns of individual performance across face-recognition tasks (Bobak, Bennetts, et al., 2016; Bobak, Dowsett, & Bate, 2016; Davis et al., 2016; Noyes et al., 2017; Robertson et al., 2016). In other words, whereas summary statistics examine the overall pattern of performance, individual performance is best seen directly from the distribution of subjects’ performance. In a review of the literature on super-recognisers, Noyes et al. (2017) point to several instances where group-level claims did not fully represent the data. Specifically, there are cases where super-recognisers perform with lower accuracy than controls, and instances when controls outperform some super-recognisers. Often, individual differences are acknowledged within experiment results; presented either in scatterplots, violin plots, or statistically with individual modified *t* test analysis (Bobak, Bennetts, et al., 2016; Bobak, Dowsett, & Bate, 2016; Davis et al., 2016; Robertson et al., 2016). However, they are often presented as an afterthought or to provide caveats to the group data. Critically, conclusions tend to be based on the group result. These group results have been reported in the media. As we will see in the current study, it is better policy to begin with individual distributions before performing any group analysis.

The second challenge in understanding how skills (e.g., body recognition and face recognition) relate to one another, is that it is difficult to distinguish “genuine ability” from the more general factor of motivation/conscientiousness. At the outset, it is reasonable to assume that motivation or conscientiousness will have some predictive value across tasks. Thus, highly conscientious people will make a sustained effort across all tasks and will likely perform better than less motivated individuals. If high performance in one task is strongly related to high performance on another task, it is unclear whether the relationship is due to skill, motivation, or to a combination of the two. This problem is particularly vexing when we observe strong correlations between face recognition performance and performance on other tasks. We should assume that motivation is part of this correlation. Thus, when processes are also related inherently (e.g., generated by similar neural mechanisms or psychological strategies), there will be strong correlation between performance on all tasks due to the underlying relationship among processes. It is difficult, perhaps impossible, to parcel out what part of the correlation is based on participant motivation versus skill. The easier case is when strong dissociation is seen between different tasks. In that case, it is reasonably easy to assume that motivation is not the entire cause of the observed correlation in task performance.

Here our goal was to determine whether a standard face-matching test (the Glasgow Face Matching Task (GFMT) short version) is an accurate screening measure of person matching. The GFMT was chosen as the screening test, because it is frequently reported in the literature as a measure of face-matching ability. Moreover, it has standardised norm scores that are available for the task (Burton, White, & McNeill, 2010). Specifically, we tested whether accuracy on the GFMT relates to identity-matching performance for face images, body images, and biological motion. In this study, we progressed through a carefully selected set of analyses that build from individual performance-level exploratory analyses to inferentially based group analysis. We first divided participants into face-recognition ability groups based on their performance on the face-matching screening task (GFMT). In the exploratory analysis, to visualise performance accuracy across our three identity-matching tasks (face, body, and biological motion), we created plots that show the full distribution of identity-matching ability for each task, colour coded by performance on the face-screening task. Multivariate analysis was also deployed to visualise the pattern of performance of individual subjects within the array of test types. Next, to compare performance of specific individuals across tasks, we created scatterplots and tested for correlation. Finally, we analysed data at a group-level using an analysis of variance (ANOVA). The visualisation methods showed that the GFMT screening predicted performance on the face task, but not on the body or biological motion task. However, group analysis supported the misleading conclusion that face recognition ability is related to performance on each of the other tasks.

## Methods

### Participants

Undergraduate students (*N* = 90, male = 12, mean age = 20 years, age range = 18–34) from The University of Texas at Dallas participated in this study in return for research credits. All participants reported normal or corrected-to-normal vision at the time of testing. The study was granted ethical approval from The University of Texas at Dallas Institutional Review Board.

### Materials and procedure

**Fig. 1:**
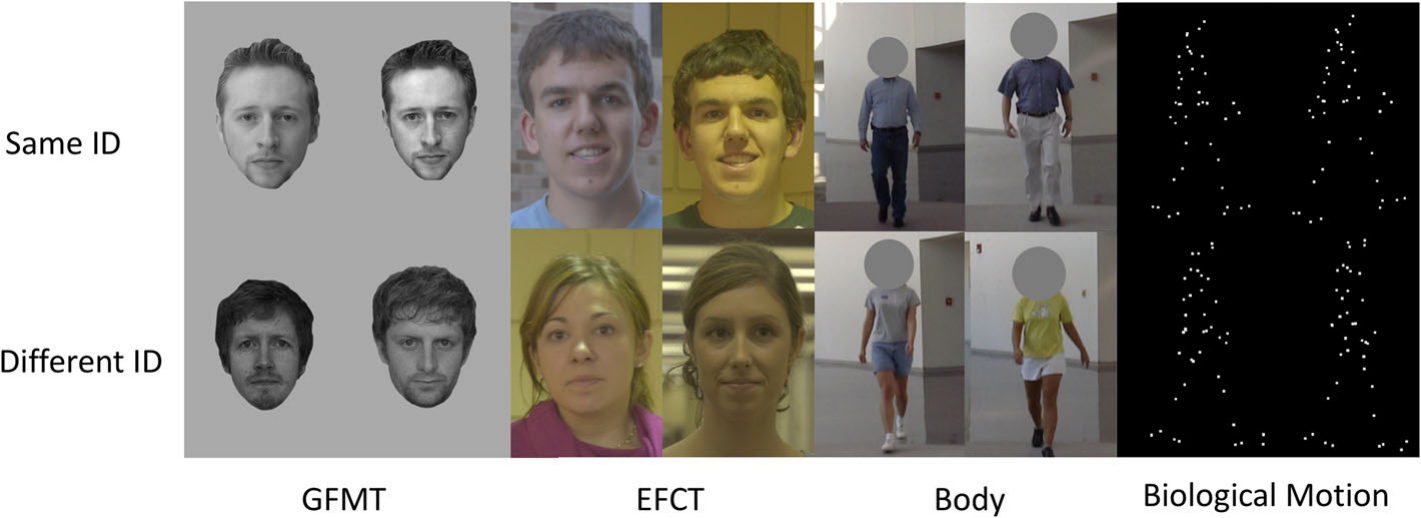
Example images from the Glasgow Face Matching Test (GFMT), Expertise in Facial Comparison Test (EFCT), Body Task and Biological Motion Task. The top row shows examples of same-identity pairs; the bottom row shows examples of different-identity pairs

All participants were tested individually on each of the following tasks. Example images for each task are shown in Fig. 1.

### Glasgow Face Matching Task 

The GFMT (short version) (Burton et al., 2010) is a standardised test of unfamiliar-face-matching ability. The task consists of the 40 most challenging face image pairs from the GFMT (full version 168 image pairs) (Burton et al., 2010). Half of the image pairs are of the same identity. Images are greyscale, front facing, taken on the same day, under similar illumination.

Our participants viewed the image pairs from the GFMT in a random order and were asked to decide whether the images in each pair were of the “same” identity or were images of two “different” people. Responses were recorded with a keyboard button press. Participants completed the task at their own pace and images remained on screen until a response was given.

### Expertise in Facial Comparison Test (EFCT) 

The EFCT (White et al., 2015) is a face-matching task consisting of 84 face image pairs (half same identity, half different identity). All image pairs were selected to be difficult items for both humans and machines to match, with different illumination conditions, and variable facial expressions. As in the GFMT, participants made decisions on the same or different identity for each EFCT image pair, with a keyboard button press. Image pairs were presented in a random order. This was a self-paced task and images remained on the screen until a response was given.

### Body task

The body-matching task was created by the authors of this paper, using footage from the Human ID database (O'Toole et al., 2005). The Human ID database contains multiple front-on walking video sessions for each identity, shot in the same location on different days. We captured 100 still-image screenshots from 100 different video clips and used these screen-shot images in our body-matching task. Each screenshot was captured when the walker reached a constant marker line to ensure that they were a constant distance from the camera. Images were edited to remove the face (covered by a grey circle), using Adobe Photoshop. Other identity “giveaway” information (e.g., exact same shoes or T-shirt) were also edited using Photoshop so that this could not be used to aid identification.

Participants viewed the body image pairs (*N* = 50) and made same or different identity decisions as was done in the body and face tasks. Image pairs were presented in pseudo-random order. Same-identity image pairs consisted of different images of the same person.

### Biological Motion Task (Baragchizadeh & O’Toole, 2017)

Point light motion videos (*N* = 20) were obtained from Baragchizadeh and O’Toole (2017), who created a database of point light videos using movement data obtained from Carnegie Mellon University’s motion capture database. All videos were normalized (to the same height, leg/arm length, etc.), therefore it was not possible to do the task based on physical properties. Where possible, points were positioned on the same XYZ coordinate for each stimulus. Half of the video items were same-identity pairs and both videos in each pair depicted movement of the same action (walk, *N* = 14; run, *N* = 1; jump, *N* = 3; box, *N* = 2). In the case of same-person pairs, two different point light videos of the same person were presented.

Participants viewed the video pairs presented simultaneously on one computer screen. The videos played three times, and then the participant responded (same or different). A response triggered the next point light video pair.

### Task order

Participants completed all four tasks in a pseudo-random order. Example images for each task are visible in Fig. 1.

## Results and discussion

### Face-recognition-ability groups

Overall accuracy on the GFMT (mean (M) = 81.99%, SE = 1.14) was consistent with standardised norms for this task (M = 81.3%, SD = 9.7) reported by Burton et al. (2010). Participants’ accuracy (percent correct) on the GFMT (short version) was used to classify participants into face-recognition-ability categories (low, medium, or high). The 30 lowest scoring participants were assigned to the low face-recognition ability group (score range 55–76). The next 30 participants were assigned to the medium level (score range 77–89) face-recognition-ability group. The top 30 participants (score range 90–100) were assigned to the high face-recognition-ability group. Face-recognition ability (with levels low, medium, and high) was used as a between-subjects variable in all subsequent analyses.

### Visualisation of individual differences

**Fig. 2:**
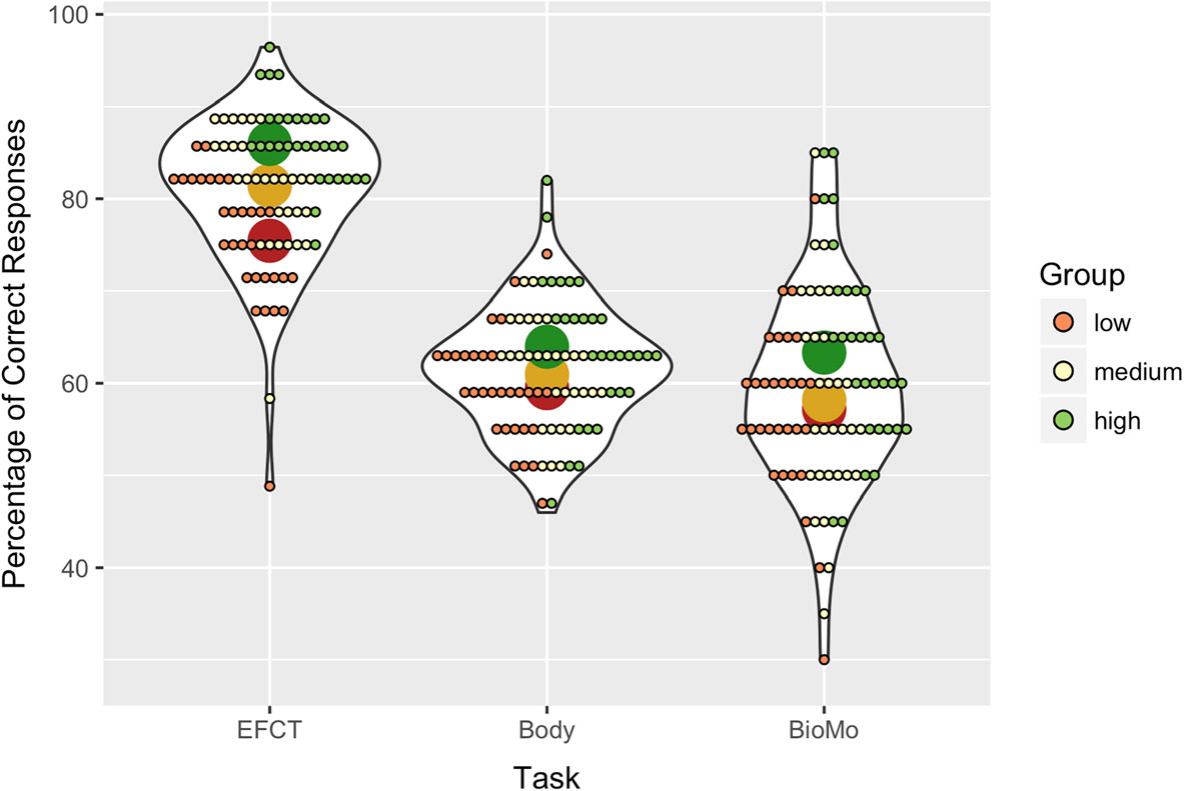
Violin plots show performance accuracy of each participant (small circles), colour coded by face-recognition ability score on the GFMT (low, red; medium, yellow; high, green) for each of the tasks (Expertise in Facial Comparison Test (EFCT) (Face), Body and BioMo (Biological Motion). Task is on the x axis, and accuracy (% correct) is on the y axis. For each task, mean performance in each face-recognition-ability group (low, red; medium, yellow; high, green) is depicted by a large coloured circle

We begin our results section with the findings that usually receive least attention in face-recognition experiments—analyses of individual differences. To visualise individual performance across tasks (EFCT, face; Body; and BioMo, biological motion), we plotted performance accuracy on a violin plot, colour coded by face-recognition-ability group (low, red; medium, yellow; and high, green) (see Fig. 2). In these plots, we can see mixing of the face-recognition-ability groups on the body and biological motion tasks. On the face task (EFCT), there is visual separation of the face-recognition-ability groups (i.e., it is easy to see the separation of the three groups). These visualisations suggest that face-matching ability (on the GFMT) is not related to performance on the body or biological motion tasks. However, from visual inspection of the graphs, GFMT performance appears to be related to performance on the EFCT.

**Fig. 3:**
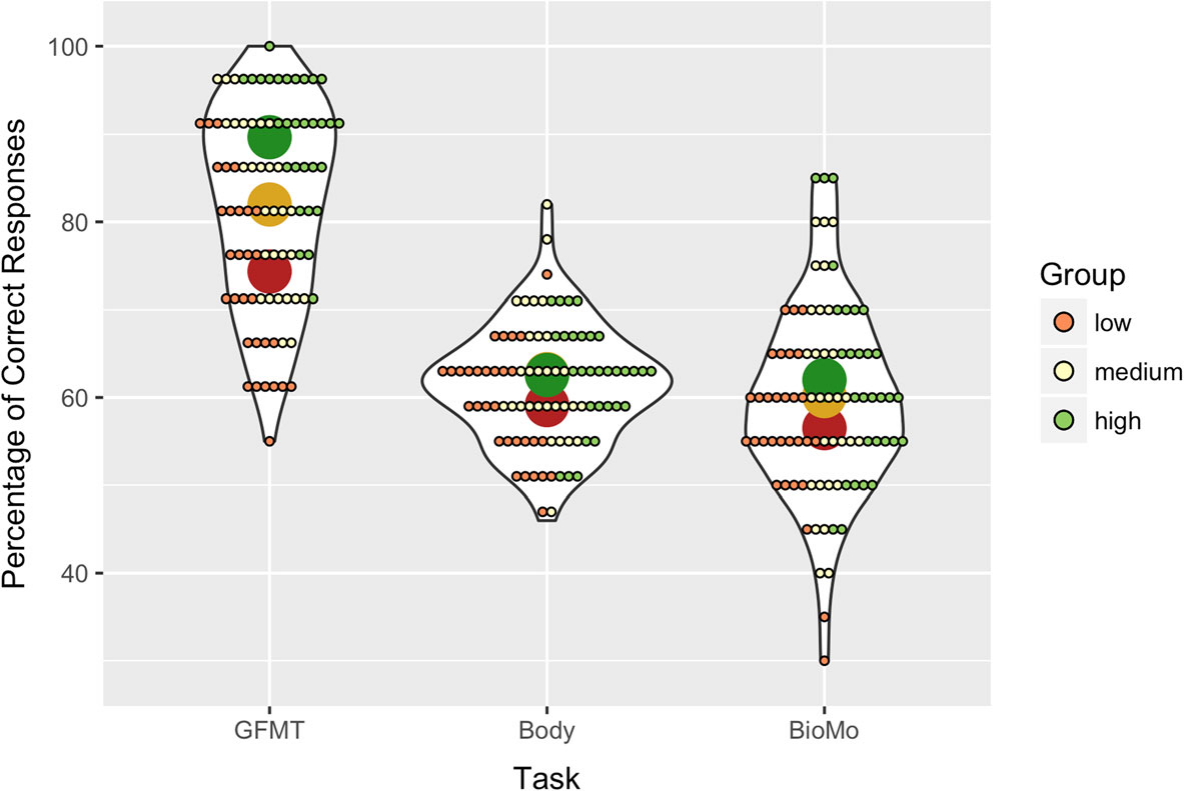
Violin plots show performance accuracy of each participant (small circles) colour coded by their Expertise in Facial Comparison Test (EFCT) ability score (low, red; medium, yellow; high, green) for tasks Glasgow Face Matching test (GFMT) (Face), Body and BioMo (Biological Motion). Task is on the x axis, and accuracy (% correct) is on the y axis. For each task, mean performance in each face-recognition-ability group (low, red; medium, yellow; high, green) is depicted by a large coloured circle

The GFMT did not appear to be an accurate predictor of performance on the body or biological motion identity-matching tasks. Given that the EFCT could also be considered as a face-based screening measure, we wondered whether performance on that task would better predict performance on the body and biological motion-matching tasks. Thus, we created a second violin plot, which grouped performance by score on the EFCT (our second measure of face-matching ability) to see if this alternative face-matching task was a better predictor of performance (Fig. 3). EFCT scores separated on the GFMT, with a large mix of performance levels on the body and motion task similar to that shown in the previous violin plot.

### Tracking individual performance across tasks

A limitation of the violin plots is that they do not track the performance of individuals across the tasks, but rather show only the locations of high, medium, and low performance for each task independently. One method for examining consistency across tasks is to apply the data stratification method used for the GFMT (low, medium, and high) to performance on each of the other task domains. In other words, to assign each participant to a performance-level group for each of the tasks, and then to assess the extent to which observers perform consistently across all tests. Thus, we categorised performance accuracy on each of the tasks into performance levels low, medium, or high by ranking performance in each task and dividing the ranked groups into the three performance categories. Next, to have an idea of where people stood, we then counted how often participants in each of the GFMT face-recognition-ability groups (low, medium, or high) fell into the corresponding ability classifications on each of the other tasks.

Only six participants in the low face-recognition-ability stratum (*N* = 30) performed with low accuracy on the other tasks for face, body, and biological motion. Out of the participants with high face-recognition-ability (*N* = 30), only six participants performed with high accuracy on all of the tasks. Thus, only 20% of people in the low face-recognition-ability group, and only 20% of people in the high-ability group, performed at the respective ability level in all three tasks.

Let us consider the implications of this result for super-recognisers. Although we did not actively seek super-recognisers for this study, GFMT scores are reported frequently for super-recognisers. There is not a clear definition of super-recogniser in the literature (c.f. Noyes et al., 2017), however super-recognisers often perform with 95% accuracy or above in the GFMT (Bobak, Dowsett, & Bate, 2016; Davis et al., 2016; Noyes & O’Toole, 2017; Robertson et al., 2016). Consequently, we created a super-recogniser stratum in our analysis that consisted of participants who scored with 95% accuracy or above on the GFMT. Among our participants, 14 met this criterion; 12 out of these 14 participants scored within the high-accuracy classification on the EFCT, and the remaining 2 scored in the medium-accuracy category: 9 participants scored within the high-accuracy classification on the body task, and 9 scored within the high-accuracy classification on the biological motion task. Critically, only 4 of the 14 top-performers were in the high-performance stratum for all of the tasks. Thus, our very best performers on the GFMT, who might meet the super-recogniser criterion in other studies, had no special advantage in the body and biological motion tasks.

**Fig. 4:**
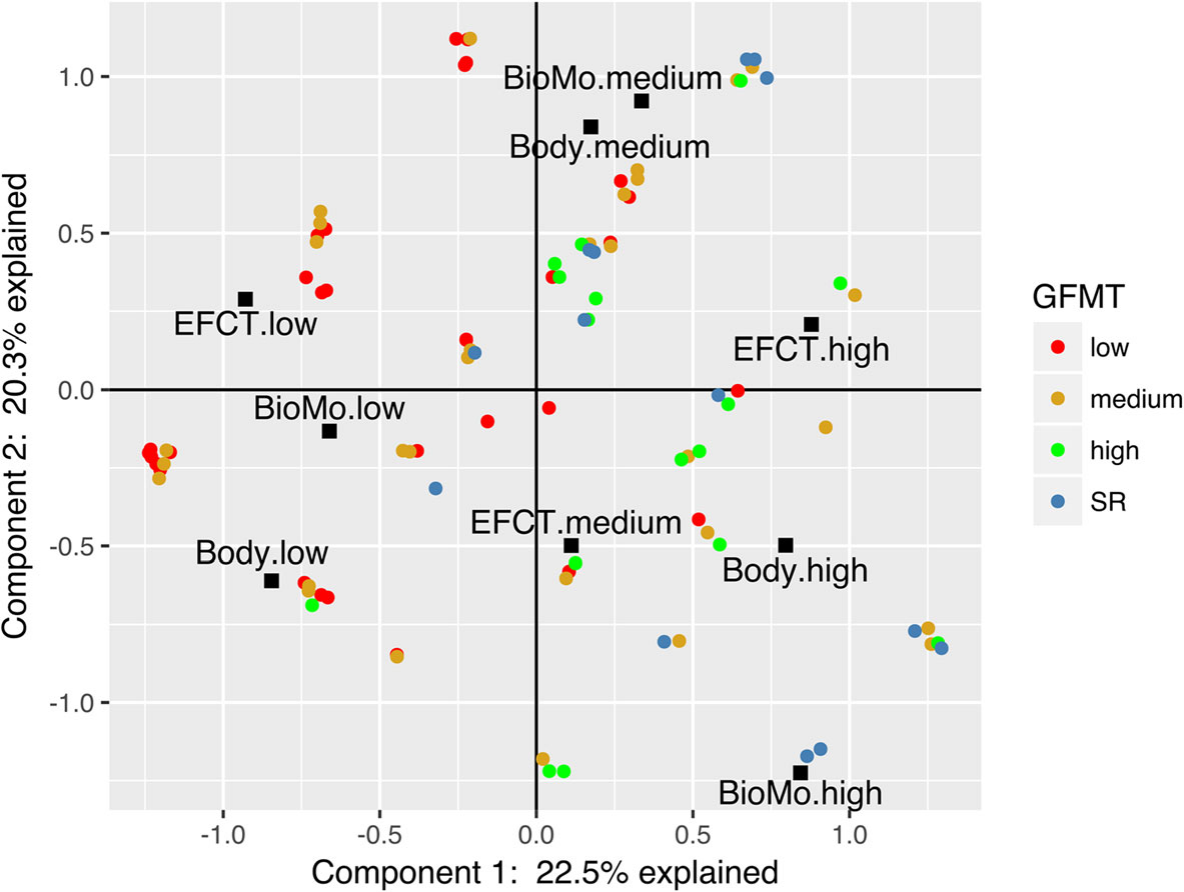
Correspondence analysis plot. Component 1 (x axis) represents performance accuracy for all tasks. This is a bi-plot which shows participants (circles) in the same plot as the levels of the variables (squares). Each circle represents the score of a single participant, colour coded by that participant’s face-recognition ability on the Glasgow Face Matching Test (GFMT) (low, red; medium, orange; high, green; SR (top-performers); blue). The similarity between the squares and the circles is shown by their angular distance relative to the origin of the space

The pattern of individual performance across the three tests can be analysed in a more systematic way with correspondence analysis, a multivariate analysis method for categorical data (Benzécri, 1973; Greenacre, 2017; Hill, Streuber, Hahn, Black, & O’Toole, 2016). Correspondence analysis is an exploratory analysis that shows relationships between variables (task performance) and observations (participants) in a shared bi-plot. This plot can be treated as a similarity space in which the patterns can be interpreted from the proximity of points in the space. In our study, this enabled us to visualise individual performance patterns across the three identification tests. We used each subject’s task performance category (low, medium, or high) for each of the three tasks (face, body, and biological motion) as input to the correspondence analysis. In other words, we are analysing the pattern of performance across tasks for individual participants. In the output we will see the consistency of individuals’ performance profiles across the tests. The output was a 6-dimensional space with its axes sorted in order of variance explained. Figure 4 shows the first two axes, which explained 42.8% of total variance. Each axis is defined by the variables with large absolute values along that axis and can be interpreted independently based on these variables. Axis 1 separates low, medium, and high performance on all tasks. Axis 2 separates extreme performance (high and low) versus medium performance. Notably, the face and body tasks behave in opposite ways to one another along axis 2. To assess whether the GFMT was an accurate predictor of performance on the three identity tasks, each participant’s data representation was colour coded by their performance on the GFMT (low, red; medium, yellow; high, green; top-performer, blue).

Crucially, there is very little accord between performance accuracy and the spread of colour- coded individual participant points. If performance on the GFMT relates to performance on the other tasks, we would expect red dots to cluster on the left, yellow dots in the middle, and green and blue dots on the right. Instead, we see a mixture of coloured dots across the horizontal axis suggesting that score on the GFMT does not predict performance on other identity-matching tasks.

### Individual differences: conclusions

The visualisations and individual data provide suggestive evidence that grouping on the GFMT (a standard face-matching task) relates to performance on a similar face-matching task (EFCT). However, these visualisations suggest that a relationship between the GFMT and the body or biological motion tasks is highly unlikely. This is reflected across all levels of face recognition ability, including super-recognisers. Next, we explore individual data further through the visualisation of scatterplots and correlation.

### Scatterplots and correlation

**Fig. 5:**
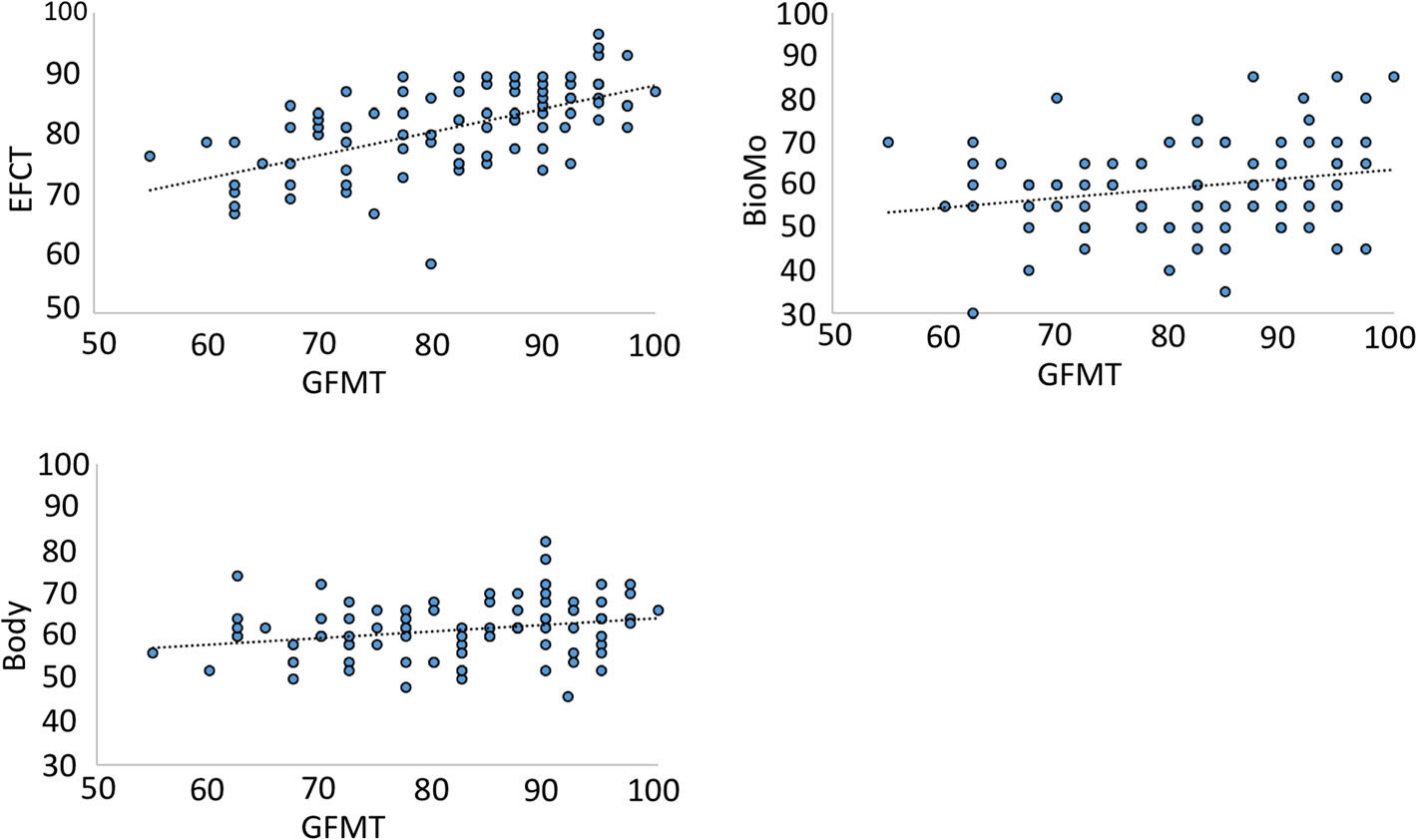
Relationship between the Glasgow Face Matching Test (GFMT) and (1) the Expertise in Facial Comparison Test (EFCT), (2) the Body and (3) the Bio Motion task

A more traditional way to show relationships is a scatterplot. We removed the factor “face-recognition-ability group” to examine point-wise ability relationships between tasks. The scatterplots for the GFMT against the EFCT appears linear, whereas the scatterplots for GFMT with the body and biological motion tests are less so (see Fig. 5).

This visualisation is supported numerically by correlation. Using the GFMT as our screening test of face-recognition ability, we calculated the Pearson coefficients for correlation between accuracy on the GFMT and accuracy on each of the other tasks. The score on the GFMT correlated significantly with scores on the face, body, and biological motion tasks. The highest correlation was between the GFMT and EFCT (*r* = .54, slope of best fit line = .77, *p* < .001). The next highest correlation was between the GFMT and the Body task (*r* = .25, slope of best fit line = .41, *p* < .05), followed by the correlation between the GFMT and the biological motion task (*r* = .23, slope of best fit line = .23, *p* < .05).

Notably, however, correlation was positive in all three of these sets of tests and was statistically significant at the standard alpha level of 0.05. In interpreting these results it is worth bearing in mind that a correlation value of *r* explains *r*^*2*^ variance in the relationship between variables. Therefore, the correlation between the GFMT and EFCT explains 29% of the relation between tests, the correlation between GFMT and Body task explains 6% of the variance between tests, and the correlation between the GFMT and the biological motion task explains 5% of the variance between tests. As a screening test, these magnitudes of the variance explained should be considered weak—especially for the relationships between GFMT and body and biological motion tests. Arguably, the relationship between the two face tests is also rather weak as a screening test. We return to this issue in the “Discussion” section.

To test whether the two body tasks were related, we tested correlation between the Body task and the biological motion task. There was no correlation between the Body task and the biological motion task (*r* = .01, *p* > .05).

### Group-based assessment of results

#### ANOVA

Finally, we computed an ANOVA, the most common statistical analysis reported in the literature on the generalisability of face-recognition skills across tasks. Data were analysed using a 3 ⨯ 3 mixed ANOVA to assess the effect of the between-subjects variable face recognition ability (high, medium, low) and the within-subjects variable task type (face, body, biological motion) on participants’ accuracy on the matching tasks. The dependent variable was the percent correct score on the face, body, and biological motion tasks. We tested for the main effects of task and GFMT face-recognition performance category, and also for an interaction between task and GFMT face-recognition performance category. A main effect of task type was expected and would simply reflect that the tasks are of different levels of difficulty.

Of critical interest to this study was whether there was a main effect of GFMT face-recognition performance category. A main effect of face-recognition ability would indicate that task performance, irrespective of task type, is related to GFMT screening-task performance. More specifically, a main effect of face-recognition ability, where there is a linear increase in accuracy from low to medium to high, would support the conclusion that the GFMT predicts performance on the other tasks. A main effect with no interaction would be the result most consistent with the use of the GFMT as a screening test. In short, the pattern of results would show a step-wise increase in each test with GFMT performance category.

However, the presence of an interaction between task type and GFMT face-recognition category would indicate a more complex relationship between these two independent variables. Namely, this indicates that face-recognition ability on the GFMT is dissociated from performance on the tasks. An interaction could indicate that some, but not all, of the tasks are predicted by the GFMT. An example of this would be if there were a linear increase in performance for one or more tasks, but not for the other(s).

**Fig. 6:**
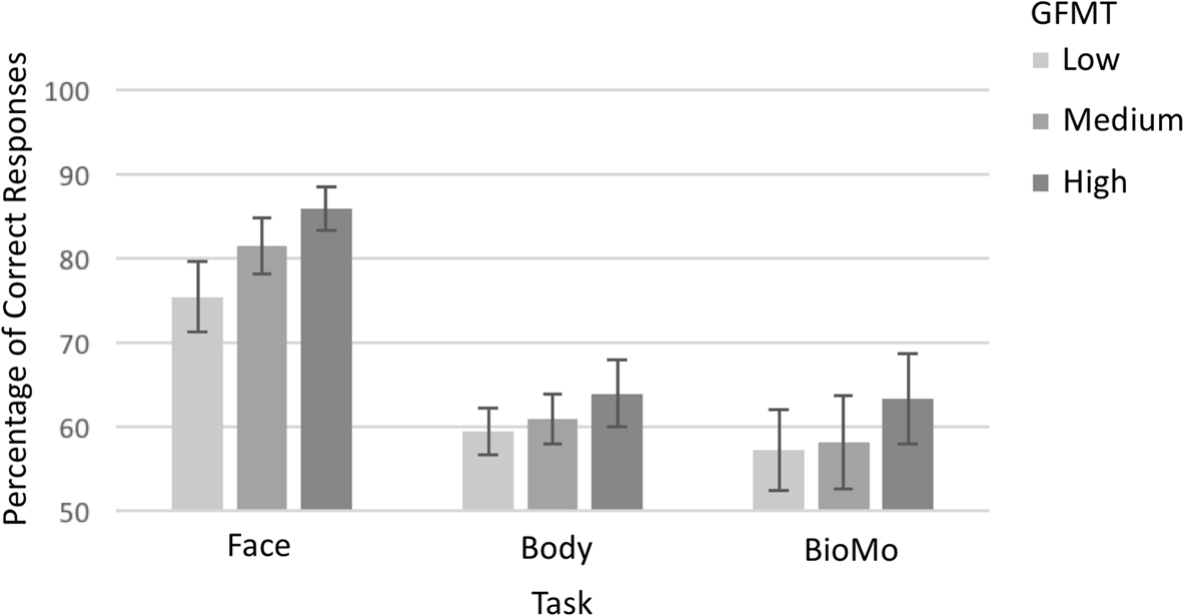
Accuracy (% correct) on each task for each Glasgow Face Matching Test (GFMT) face-recognition-ability level (low face recognition, medium face recognition, high face recognition). Error bars show standard deviation from the mean

As expected, there was a significant main effect of task type (*F*(2, 174) = 201.66, *p* < .001, *ηp*^*2*^ = .70). Accuracy was highest for face matching (M = 80.95%, SE = .72) followed by body matching (M = 61.46%, SE = .68) with lowest performance on the biological-motion-matching task (M = 59.56%, SE = 1.12). There was also a significant main effect of face-recognition ability (high, medium, low) on performance accuracy on the tasks (*F*(2, 87) = 16.96, *p* < .001, *ηp*^*2*^ = .28). Highest overall task accuracy was achieved by the high face-recognition-ability group (M = 70.71%, SE = .91), followed by the medium face-recognition-ability group (M = 66.51%, SE = .91), and then the low face-recognition-ability group (M = 64.72%, SE = .91). Importantly, there was no interaction between face-recognition ability and task type (*F*(4, 174) = 1.48, *p* = .21, *ηp*^*2*^ = .03), showing that the pattern of increased accuracy with increased face recognition applied to all tasks (See Fig. 6).

For completeness, this analysis was repeated using *d′* as the measure of accuracy. The pattern of results with *d′* was identical to that found with percent correct. The full breakdown of *d′* and criterion results are presented in the Appendix.

### Group analysis: conclusions

In summary, analyses that involved summary statistics based on overall data (correlation) and group data (ANOVA) produced statistically significant results that are generally consistent with the conclusion that performance on the face-recognition screening task (GFMT) predicts, or is related to, performance on the other person-matching tasks. If group-based data had been computed blindly, this would indeed be the conclusion drawn. However, individual data (visualised in the first stage of the analyses) show clearly that any group-level effects of face-recognition ability on task performance must be interpreted with caution. Whilst face-recognition ability predicts performance at a group level, the large individual differences in the relationship between face-recognition ability and performance on the body and biological motion task are strongly indicative of the conclusion that the GFMT should not be used as a screening test for the more general task of person matching from bodies and biological motion.

## General discussion

This study is the first to test the relationship among person-matching skills from the face, body, and biological motion. The face, body, and biological motion all contribute to person identification (Hahn et al., 2016; O'Toole et al., 2011; Rice et al., 2013; Robbins & Coltheart, 2015; Simhi & Yovel, 2016; Stevenage et al., 1999; Troje, 2005). We extended previous investigations with prosopagnosic individuals (Biotti et al., 2017) to include the general population with different face-recognition abilities. We also included biological motion as a skill of interest. Taken as a whole, our data offer only weak support for the claim that performance at face recognition predicts performance on person matching, either from bodies or biological motion. The weak nature of the effects is clear from the scatter of individual performance in the violin plots and in the correspondence analysis. Thus, despite the fact that the group analyses showed a pattern of performance that did not violate expectation for the claim, a careful look at the individual data indicates that group data analysis is not appropriate for the data. We consider why these tests are inappropriate as we proceed. Can the GFMT be used as a predictor for face recognition in another test (e.g., EFCT)? The results here are less certain and would depend on the level of accuracy required with such predictions. It is clear that the GFMT predicted performance on the EFCT with greater accuracy than it predicted performance for the body and biological motion tasks. It is also clear that prediction accuracy exceeded chance. Arguably, however, the individual differences observed suggest that the use of the GFMT would lead to substantial numbers of prediction errors at the level of individual participants.

From a more theoretical point of view, the dissociation we found between ability to identify people from face images, body images, and biological motion, is not entirely consistent with the neural hypotheses we considered. Based on neural data, we predicted that face and body recognition abilities may be linked (through ventral stream processing), but face and biological motion recognition might be dissociated (due to processing of motion in the dorsal stream). Our results are inconsistent with this hypothesis, because both body and biological motion recognition ability was dissociated from face-recognition ability. Our results are also inconsistent with the holistic processing account. The majority of previous data on processing strategies suggests that holistic processing characterises face, body, and biological motion perception. We have no evidence against this; however, our data would not point to holistic processing supporting shared person recognition abilities across domains.

Returning to the applied question of whether the GFMT, or any recognition test, is a suitable candidate as a screening test, the present study points to several important considerations. First, we must propose an appropriate goal for the level of prediction accuracy we require for a screening task that will be used in an applied setting. The results of our study highlight that the literature as a whole may be relying on statistical methods that do not match the goals of evaluating screening tasks. Inferential statistical tests determine whether the strength of a relationship between variables exceeds a chance relationship. This is a rather low bar for the question at hand, and it must be decided whether this is an appropriate threshold for screening individuals who will be providing evidence for life-altering judicial hearings. There is a difference between our expectation for high-accuracy predictions that may be dictated by the serious nature of the task and the group test criterion of “better than chance”. Therefore, it is not surprising that conclusions based on group versus individual data have diverged in this literature (Biotti et al., 2017; Bobak, Bennetts, et al., 2016; Davis et al., 2016; Noyes et al., 2017).

Second, and a related issue, it has been known for some time that analyses based on individual differences do not always converge with analyses based on group data. An open question is how to address this issue in the context of evaluating a screening test. If correlation testing or ANOVA are used as the main analysis method in a study, the conclusions will be based on comparisons that are made against chance performance. If other methods are to be used, the conclusions made from group and individual data will align if criteria for results are set at the outset. For example, if above-chance results are deemed acceptable for the task at hand, group data can be used to make conclusions, and individual data used to support a deeper understanding of the results. Alternatively, if stricter criteria are implemented, and if the group and individual data contradict one another, then the individual data and regression analysis must inform conclusions. The present study highlights the importance of visualising individual data in research. We suggest that violin plots, or equivalent, be used to visualise individual data as standard practice.

In terms of moving forward in applied scenarios, a prediction criterion that is appropriate for the task at hand must be set for screening tests. The goal, although implausible in practice, is ideal prediction. Instead, different criterion levels may be suited to different applications, because these applications have different consequences. High criteria should be set for the recruitment of a Federal Bureau of Investigation (FBI) forensic image examiner, whose identity decisions will be used as evidence in court. Lower criteria may be adequate for job roles with less critical consequences. For jobs that involve low-impact identity decisions, the results of group-based screening analysis may be sufficient. When a judicial verdict is made by a screened identifier, the screening criterion should be filtered to the court, to the media, and to the public.

Turning to the applicability of results for super-recognisers, our results point to several important findings. Although we did not actively recruit super-recognisers in our study, 14 of our high performers on the GFMT scored with an accuracy that might meet super-recogniser criteria in other studies. We refer to these 14 individuals as “top-performers”. Our top-performers on the GFMT generally performed well on the EFCT, but like participants in all other GFMT face-recognition-ability categories, their performance varied across the body and biological motion task. This shows that the top-performers did not have “super” abilities on the body and biological motion task.

On a theoretical note, the field has not yet converged on whether face-recognition ability lies on a spectrum, or whether super-recognisers are a “special” group of face-recognisers. A recent review of the literature, however, comes to the conclusion that super-recognisers are best thought of as the top tail of the distribution of normal face-recognisers (Noyes et al., 2017). In that review, the authors note that there is no evidence yet to suggest that super-recognisers are qualitatively different than normal face-recognisers. The results of the present study are consistent with super-recognisers as part of the general population. Specifically, the distribution of face-recognition ability was similar to that seen for the other person-perception tasks and followed a normal distribution (see Fig. 2). Our findings support that face-recognition ability, like all other person-perception abilities, falls on a normally distributed spectrum of ability.

The findings of this study can be used to inform future research. We argue that future studies must first address the issue of consistency of performance for same-domain tests. This study shows some consistency in performance across two different face-recognition tasks taken on the same day. It is not yet known whether this consistency will hold across time. If same-domain tasks are consistent across time, then this would support a case for implementation of domain-specific screening tasks to assess each skill type necessary for a specific person-identification job.

## Conclusion

In conclusion, we found no convincing evidence that face-recognition ability generalises to body and biological motion recognition. However, we found a moderate predictive relationship between two tests of face-matching ability. We highlight the challenge of dealing with discrepancies between the conclusions one would make from individual versus group-based analyses. The present results point to the use of individual differences to inform how, or indeed, whether to apply group analyses—rather than to use individual differences as a caveat to the conclusions made with group analyses.

## Appendix

### Accuracy Measure: *d′*

For completeness, accuracy was also assessed using *d′*. A response was recorded as a “hit” if the participant correctly responded “same” when the answer was “same”, and “false alarm” if the participant responded “same” when the answer was “different”. We conducted 3 ⨯ 3 ANOVA, as aforementioned, this time using *d′* as the measure of accuracy. The pattern of results with *d′* was identical to that found with percent correct. Specifically, accuracy varied with task type (*F*(2, 174) = 242.87, *p* < .001, *ηp*^*2*^ = .74). The highest accuracy was achieved for faces (M = 2.01, SE = .06), followed by the body task (M = .63, SE = .04), and then the biological motion task (M = .53, SE = .06). GFMT face recognition category affected performance (*F*(2, 87) = 16.65, *p* < .001, *ηp*^*2*^ = .28), again with the groups ordered as high (M = 1.31, SE = .55) better than medium (M = 1.00, SE = .55), better than low (M = .87, SE = .55). Task and GFMT face-recognition category did not interact (*F*(4, 174) = 2.11, *p* = .08, *ηp*^*2*^ = .04).

### Criterion

To measure response bias, criterion was analysed by an ANOVA with the same design. Here, a positive response bias indicates a tendency to respond “different” identity, and a negative response bias indicates a tendency to respond “same” identity. There was a main effect of task type on criterion (*F*(2, 174) = 54.79, *p* < .001, *ηp*^*2*^ = .39). Participants were most likely to make different person identity decisions for the face task (M = .35, SE = .04), showed no response bias for the biological motion task (M = .00, SE = .03), and were more likely to respond same identity in the body task (M = −.08, SE = .34). Criterion did not vary across face-recognition category (*F*(2, 87) = 1.85, *p* = .16, *ηp*^*2*^ = .04). There was no interaction between task and face-recognition category (*F*(4, 174) = .29, *p* = .88, *ηp*^*2*^ = .01).
